# Taxonomy of *Cyrtomorphus* Lacordaire (Coleoptera, Erotylidae, Tritomini) from China

**DOI:** 10.3897/zookeys.886.37991

**Published:** 2019-11-05

**Authors:** Na Jia, Xu Su, Jing Liu, Weicheng Lu, Guodong Ren, Jing Li, Yuyu Wang, Yanchen Zhao

**Affiliations:** 1 College of Plant Protection, Hebei Agricultural University, Baoding 071001, China Hebei Agricultural University Baoding China; 2 College of Life Science, Hebei University, Baoding 071001, China Hebei University Baoding China

**Keywords:** Cucujoidea, description, key, new species.

## Abstract

In this paper, a key to separate the Chinese species of genus *Cyrtomorphus* Lacordaire is provided for the first time. A new species, *Cyrtomorphus rufobrunneus***sp. nov.**, is described and illustrated, and one species, *Cyrtomorphus connexus* Gorham, 1896 is newly recorded to China.

## Introduction

The genus *Cyrtomorphus* was established by Lacordaire (1842) for *Cyrtomorphus pantherinus* Lacordaire, 1842, which is mainly distributed in the Oriental region. This genus can be distinguished from other genera in the tribe Tritomini Curtis, 1834 by the following characters: body oval or broadly ovate, distinctly convex on the dorsum, antennomere XI small, compound eye small and finely facetted, lateral walls of oral cavity forming flattened plates; males of some species with a row protuberance on inner surface of middle tibia.

The genus *Cyrtomorphus* (Coleoptera, Erotylidae, Tritomini) included 35 species worldwide (Boyle 1956; Delkeskamp 1981; Chûjô and Chûjô 1990; Alvarenga 1994; Wegrzynowicz 2007) with five species known from China. *Cyrtomorphus yunnanus* Mader was described from Yunnan (Mader 1937). *Cyrtomorphus duodecimmaculatus* Araki was described from Taiwan (Araki 1941). Later, *Cyrtomorphus chinensis* Mader and Cy*rtomorphus duxoides* Mader were described from Fujian (Mader 1955). *Cyrtomorphus liui* Chûjô was described from Taiwan (Chûjô 1967).

Before the present study, all taxonomic research on *Cyrtomorphus* in China was at least 50 years ago. But recently, specimens of *Cyrtomorphus* were collected and deposited in Museum of Hebei University (MHU). One new species was found among 52 specimens, *Cyrtomorphus rufobrunneus* sp. nov., from Guizhou Province, and is described and illustrated. A second species, *Cyrtomorphus connexus* Gorham, 1896, is recorded from China for the first time. Additionally, a key to the seven species from China is included.

## Materials and methods

The abdominal segments and the genitalia were detached from the body after softening in hot water. Male or female genitalia were put in 5% NaOH boiling solution for 5 minutes and then cleaned with distilled water. Morphological characters were observed using a Nikon SMZ800N stereomicroscope. Photographs were taken with Cannon Eos 5D Mark III camera and Canon EF 100mm f/2.8L Macro IS USM lens and modified with Adobe Photoshop CC 2016. The species distribution was inferred from examined materials and published records. The specimens studied were deposited in MHU.

## Taxonomy

### Key to the species of *Cyrtomorphus* from China

**Table:** 

1	Pronotum coloration uniformly black	***C. yunnanus* Mader**
–	Pronotum coloration not monochrome, with color pattern	**2**
2	Pronotum, except for the outer margin, brownish black	***C. rufobrunneus* sp. nov.**
–	Pronotum with two or more patches	**3**
3	Four black spots on pronotum	***C. connexus* Gorham**
–	Two black flecks in the pronotum	**4**
4	Elytron with four black marks	**5**
–	Elytron with five black marks	**6**
5	Pronotum with two spots on basal margin	***C. chinensis* Mader**
–	Pronotum with two spots on frontal margin	***C. duxoides* Mader**
6	Elytron suture with two united patches	***C. liui* Chûjô**
–	Elytron suture without united patches	***C. duodecimmaculatus* Araki**

#### Cyrtomorphus rufobrunneus

Taxon classificationAnimaliaColeopteraErotylidae

Jia & Li sp. nov.

A733691B-D75F-576C-8971-643D55134B0C

http://zoobank.org/4AF1346E-6111-4E4E-B180-954869AE2800

Figures 1–2 , 3–14

##### Type material.

**Holotype.** CHINA: ♂; fully matured beetle (MHUB01223). Guizhou, Libo County, Maolan National Nature Reserve, 25.3112N, 108.0761E, alt: 755m, 20 July 2015, Caixia Yuan leg.

**Paratypes.** CHINA: 2 ♂♂, 2 ♀♀, (MHUB01224–MHUB01227), same data as for holotype.

##### Diagnosis.

Body oval convex, widest at base of elytra. General color red-brown, legs and antennae black, antennomere I and II paler, pronotum except the outer margin brownish black, each elytron with a brownish-black band. Clypeus with the anterior border emarginated, clypeofrontal sulcus completed. Antennomere III about 1.5 times as long as IV; relative lengths of antennomeres II–XI: 40: 100: 62: 55: 55: 52: 50: 125: 105: 70. Terminal segment of maxillary palpus asymmetrical triangular, nearly half as long as wide. Pronotum with anterior border directly opposite head, slightly projecting forwards in the middle. Pro-, meso-, and metacoxal lines present.

##### Description.

**Figures 1, 2.**
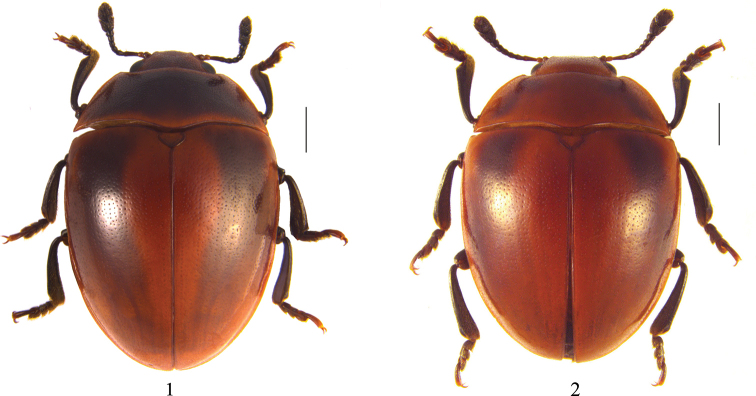
Dorsal habitus of *Cyrtomorphus rufobrunneus* sp. nov. **1** holotype **2** paratype (MHUB01224). Scale bars: 1.00 mm.

Length: 7.0–9.5 mm, width: 4.5–6.0 mm. Body oval, almost hemispherical, convex, shiny and smooth, widest at base of elytra, general color red-brown, legs and antennae black (antennomeres I–II paler). Pronotum except the outer margin brownish black. Markings on pronotum can differ depending on age; some with a broad, lateral, brownish-black band that almost reaches front and basal margin. Scutellum red-brown, both sides darker. One brownish black band on each elytron, with the color becoming paler from base to apex (Figs 1, 2).

**Figures 3–14.**
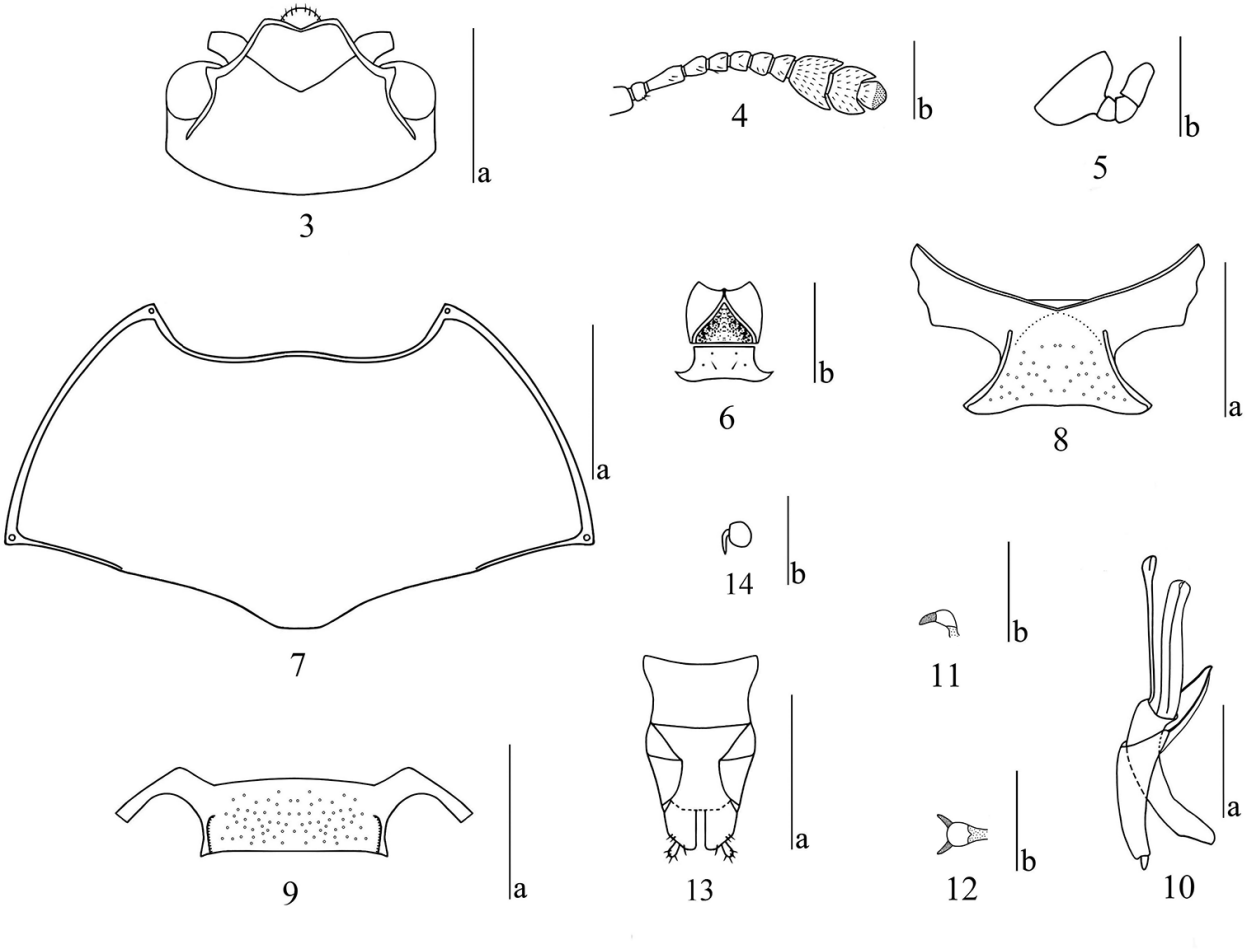
*Cyrtomorphus rufobrunneus* sp. nov. **3** head **4** antenna **5** maxillary palpus **6** mentum **7** pronotum **8** prosternum **9** mesoventrite **10** male genitalia in lateral view **11, 12** lateral and dorsal views of anterior end of male flagellum **13** female genitalia in ventral view **14** female spermatheca. Scale bars: 1 mm (**a**), 0.5 mm (**b**).

***Head*** (Fig. 3) small; strongly and sparsely punctured on vertex, vertex puncture size approximately 2 times facet diameter, separated by 2–3 diameters. Clypeus strongly and closely punctured, clypeal puncture size same as facet, nearly coalescing; anterior border obviously emarginated; clypeofrontal sulcus completed. Eyes small, moderately prominent and finely faceted; interocular distance 0.70 times width of head. Antennae (Fig. 4) short, extending to basal three-fourths of pronotum, with short, pale-yellow setae; antennomere II round; antennomere III nearly 1.6 times as long as IV; antennomeres IV to VIII subequal, antennomeres VII and VIII slightly wider than VI; antennomere IX bowl-shaped; antennomere X broadly crescent-shaped; antennomere XI small, almost scallop-shaped, half surrounded by antennomere X; relative lengths of antennomeres II–XI: 40: 100: 62: 55: 55: 52: 50: 125: 105: 70. The terminal segment of maxillary palp (Fig. 5) subtriangular, asymmetrical in lateral view, length about 0.5 times as long as width. Mentum (Fig. 6) with pentagonal plate, both sides margined, with middle area depressed; submentum finely and sparsely punctured, with a few setae.

***Pronotum*** (Fig. 7) transverse, widest at basal (pronotum length/width ratio 0.52); lateral border slightly curved, margined; anterior border opposite head, with slightly forward projection in the middle, with fine margin; basal border weakly sinuate, with margins on both sides. Punctures on pronotum similar to vertex, puncture size 2 times facet, separated by 2–3 diameters on both sides, decreasing in size and density toward median area, disc puncture size same as facet, separated by 3–4 diameters. Anterior angle and posterior angle projecting, each with a pore.

***Scutellum*** heart-shaped, with fine punctures.

***Elytra*** widest at base, EL/EW ratio 1.1, gradually narrowing to apex. Each elytron with six or seven indistinct striae; intervals finely punctured.

***Prosternum*** (Fig. 8) with textured surface laterally; fine and sparse punctures medially, with golden setae. Prosternal process broad, produced to an indistinct point anteriorly, and emarginated at posterior border; prosternal lines straight, converging and extending to the front edge of coxae.

Mesoventrite (Fig. 9) broad, each side with a shallow depression, sternum with coarse punctures, size 2 times facet.

Metaventrite coarsely and sparsely punctured in the middle, without punctures on each side of base, with a longitudinal depression on posterior 5/6; coxal lines extending to basal one-third of metaventrite.

***Abdomen*** with coarse and dense punctures laterally, puncture size 2.5 times facet; punctures smaller medially, 0.5 times facet, with short golden setae; long coxal lines on first ventrite nearly attaining posterior border.

***Male genitalia*** (Fig. 10) with median lobe weakly curved, narrowing from one-half to two-thirds, then gradually widening to 5/6; from here narrowing to a blunt point; median strut as long as median lobe; flagellum short, length = 0.85× median lobe length; sclerite at anterior end of flagellum as in Figures 11, 12.

***Female genitalia*** (Fig. 13) with narrow styli at apex of coxite, covered with setae at apex. Female spermatheca (Fig. 14) almost round.

Middle tibia of male without a row of protuberances on the inner surface.

##### Distribution.

China: Guizhou.

##### Remarks.

*Cyrtomorphus rufobrunneus* sp. nov. is similar to *Cyrtomorphus duodecimaculata* Araki, 1941 in its form and body color. The new species can be distinguished by each elytron bearing a brownish-black band, the pronotum having a projecting posterior angle, the prosternal lines straight, and males without a row of protuberances on the inner surface of middle tibia. In contrast, *Cyrtomorphus duodecimaculata* has five black marks on each elytron, the pronotum has obtuse posterior angles, the prosternal lines curved, males have a row protuberances on the inner surface of the middle tibia.

##### Etymology.

The species is named for the red-brown body color.

#### Cyrtomorphus connexus

Taxon classificationAnimaliaColeopteraErotylidae

Gorham, 1896

66BAD751-233C-570A-936C-129F12174914

Cyrtomorphus connexus Gorham, 1896: 286.

##### Material examined.

CHINA: 3♂♂, 4 ♀♀, Yunnan, Mengla County, Mohan Town, 21.0624N, 102.3077E, 3–4 July 2006, Guodong Ren, Wenjun Hou & Yalin Li (MHU) leg.

##### Distribution.

New for China (Yunnan), Burma.

##### Comparative notes.

**Figures 15, 16.**
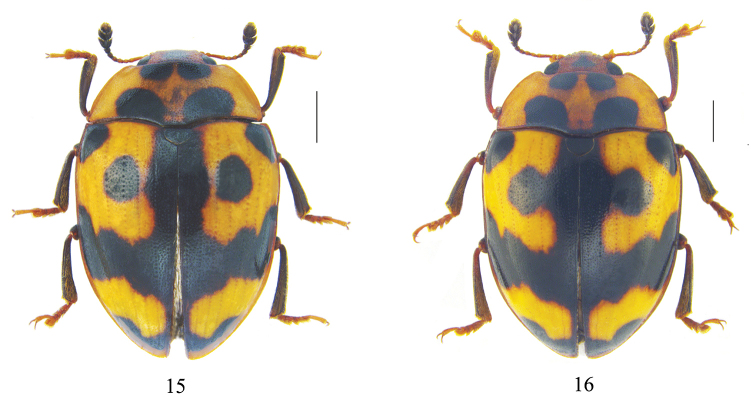
Dorsal habitus of *Cyrtomorphus connexus* Gorham, 1896 **15** elytra with black spot enclosed **16** elytra with open black spot. Scale bars: 1 mm.

The original description was based on the unique type specimen from Karen Hills, Burma (Gorham 1896). Considerable interspecific variability of *C. connexus* was observed in external characters such as body size, coloration, punctation, and stripes of the head, pronotum, and elytra (Figs 15, 16). It is recorded here from Yunnan Province for the first time. Some specimens are similar to the type specimen, while others differ slightly with the black spot on elytra enclosed (Fig. 16). Arrow (1925) also pointed out that the black markings may be less united.

## Supplementary Material

XML Treatment for Cyrtomorphus rufobrunneus

XML Treatment for Cyrtomorphus connexus
